# Perioperative management of paraganglioma and catecholamine-induced cardiomyopathy in child– a case report and review of the literature

**DOI:** 10.1186/s12871-017-0433-0

**Published:** 2017-10-17

**Authors:** Xixi Jia, Xiangyang Guo, Qing Zheng

**Affiliations:** Department of Anesthesiology, Peking University Third Hospital, 49 North Garden Rd, Haidian District Beijing, 100191 People’s Republic of China

**Keywords:** Paraganglioma, Catecholamine-induced cardiomyopathy, Child, Norepinephrine, Anesthetic management

## Abstract

**Background:**

Paragangliomas are catecholamine-secreting tumors of the paraganglia. Perioperative mortality of children with paraganglioma is high, but preoperative therapy and anesthetic management of paraganglioma resection are controversial in children. The literatures on catecholamine-induced cardiomyopathy are limited to several case reports,with few reports of studies on children.

**Case presentation:**

Here we report the anesthetic management of a child with paraganglioma and catecholamine-induced cardiomyopathy, and the possible perioperative anesthesia problems of the paraganglioma resection are discussed.

**Conclusion:**

Preoperative and intraoperative anesthetic management of Pheochromocytomas children should follow the same principles as for adults, The most important aspects are the control of blood pressure liability and maintenance of adequate blood volume. Pheochromocytomas patient may have cardiomoyopathy due to myocardial toxicity of excessive circulating catecholamines level. The perioperative management of catecholamine-induced cardiomyopathy should include lowering sympathetic activation by means of α-and β-adrenergic receptor blocker and diuretics administration in case of volume overload.

## Background

Pheochromocytomas and paragangliomas are catecholamine-secreting tumors of the paraganglia. The tumors are found mainly in the adrenal medulla, known as pheochromocytomas. Those found outside the adrenal gland are called paragangliomas. Clinical expressions of them are highly variable, ranging from asymptomatic to life-threatening systemic hypertension and conduction disturbances [1]. The patients may have cardiomyopathy caused by myocardial toxicity of excessive catecholamines, such as norepinephrine, epinephrine and rarely dopamine, etc. [2, 3]. The tumors are mostly benign of chromaffin cells of the adrenal medulla or of extraadrenal paraganglia. In about 10% of cases, they can be malignant [4].

In this article, we report the anesthetic management of a young child with paraganglioma and catecholamine-induced cardiomyopathy,and the possible perioperative anesthesia problems of the resection of paraganglioma are discussed.

## Case presentation

**Fig. 1:**
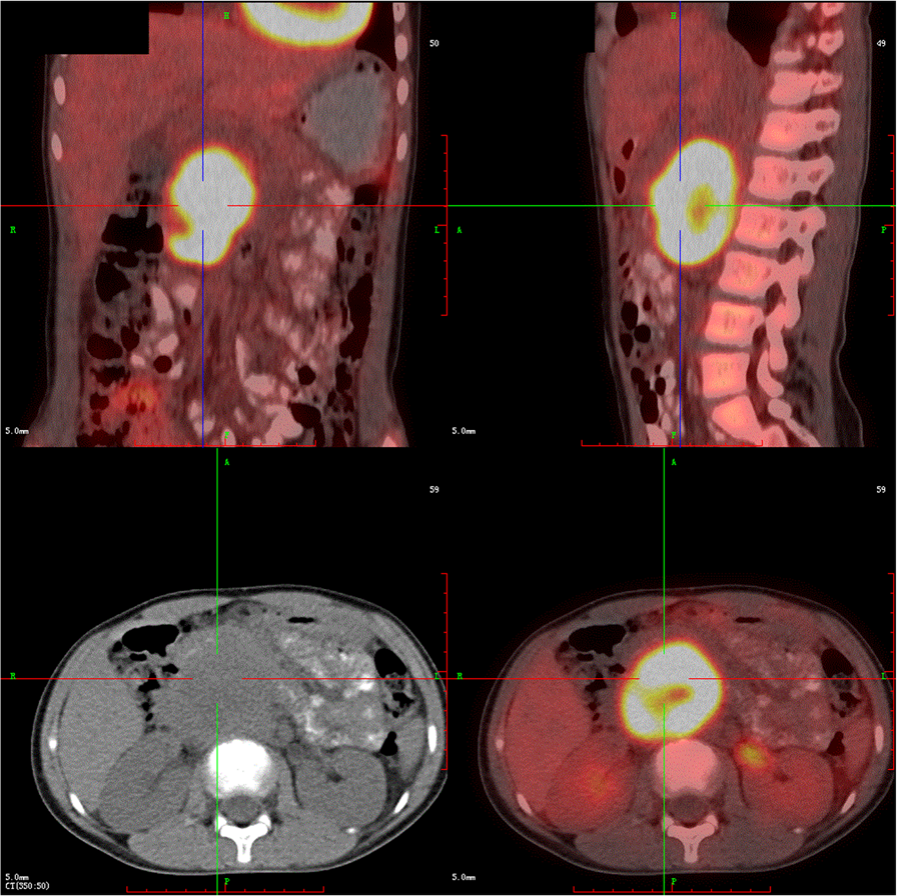
A 5 × 4 cm extra adrenal paraganglioma encircling the abdominal aorta

**Fig. 2:**
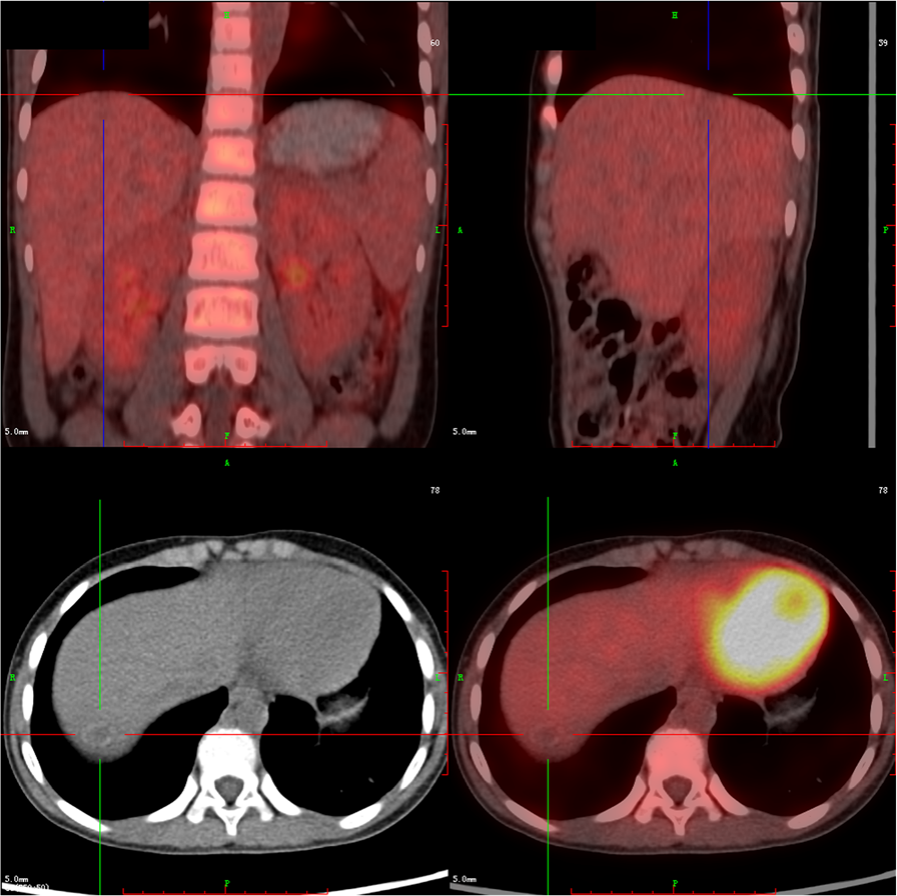
A 2 × 1.4 cm metastatic tumor in liver

A 9-year-old girl (130 cm,33 kg) was scheduled for open resection of a right 5 × 4 cm extra adrenal paraganglioma encircling the abdominal aorta (Fig. 1)and a 2 × 1.4 cm metastatic tumor in liver(Fig. 2). Her medical history included dyspnea on exertion and persistent hypertension(150–160/90-100 mmHg).Her serum adrenaline, noradrenaline and dopamine levels were 0.617 pmol/ml(normal,0.05–1.39 pmol/ml),97.417 pmol/ml(normal,0.51–3.62 pmol/ml)and0.046 pmol/ml(normal,0.07–0.68 pmol/ml). The coagulation studies and NT-proBNP level were normal, but a transthoracic echocardiogram demonstrated mild mitral regurgitation and left ventricular dilation(LVEDV,43 mm) with an estimated LVEF of 49%. The diagnosis of paraganglioma and catecholamine-induced cardiomyopathy was made.

Preoperatively, her blood pressure was 120–130/80-90 mmHg, well controlled on phenoxybenzamine(20 mg/Q12h orally).Fluid(2000 ml/d)had been given for 4 weeks to restore the blood volume. On the morning of the operation, her supine blood pressure was 120/70 mmHg with heart rate 80–100 beats/min. Midazolam 1 mg was administered intravenously as premedication. On arriving in the operating room, she acted calm and cooperative. Left radial arterial blood pressure monitoring was established under local anesthesia before anesthesia induction. Simultaneous measurements of cardiac output and stroke volume variation as determined by the FloTracTM device (Edwards Lifesciences)were also recorded. General anesthesia was induced with propofol 60 mg,sufentanyl 30 μg and rocuronium 30 mg.The trachea was intubated without an increase in blood pressure. An invasive central venous catheter was placed via the right internal jugular vein. Anesthesia was maintained with inhaled sevoflurane in oxygen, combined with sufentanyl and rocuronium injected intravenously. The operation was performed in the supine position with her systolic pressure stabilized at 100 mmHg, central venous pressure at 8 mmHg and heart rate at 90 beats/min. When the skin incision was made, her blood pressure kept stable without the infusion of vasodilators. Before the tumor manipulation, we started a continuous intravenous infusion of 0.5 mg/h of sodium nitroprusside. Just after the start of tumor manipulation, her blood pressure and heart rate exceeded 180/110 mmHg and 120 beats/min respectively, and a bolus injection of phentolamine 2 mg was followed. Blood pressure and heart rate decreased to 150–170/80-100 mmHg and 110 beats/min with the continuous infusion of sodium nitroprusside at 2 mg/h. Removal of the right-sided paraganglioma was difficult, immediately after the resection of the tumor, blood pressure and heart rate decreased to 110/60 mmHg and 100 beats/min, managed with the continuous infusion of sodium nitroprusside at 1.2 mg/h. According to the estimated blood loss (approximately 400 ml), a combination of two unit packed red blood cells and 200 ml fresh frozen plasma were transfused and CVP increased from 6 mmHg to 8 mmHg.During the dissection of the metastatic tumor in the liver, blood pressure and heart rate increased to 150/90 mmHg and 120 beats/min again. Hemodynamics could be satisfactorily controlled with a further 2 mg intravenous phentolamine. Before the resection of the metastatic tumor, continuous infusion of sodium nitroprusside was stopped, but systolic blood pressure decreased rapidly to approximately 70 mmHg after removal of the tumor. The status was corrected by continuous infusion of norepinephrine at 0.05 μg/kg/min to maintain an reasonable level in systolic blood pressure to 100 mmHg.At that time,the CVP was 7 mmHg and HR was 110 beats/min(Table 1). Blood glucose remained between 9 and 11 mmol/L under anesthesia and body temperature was 36.5–37.3 °C. The patient was transferred to the ICU with trachea intubated and a continuous infusion of norepinephrine at 0.12 μg/kg/min. The total operative duration was 5 h and 29 min. The blood loss was 600 ml and the total quantity of fluid administered was 2800 ml(1700 ml crystalloid solutions, 500 ml colloidal solutions, two unit packed red blood cells and 200 ml fresh frozen plasma).

**Table 1:** Hemodynamic parameters and arterial blood gas analysis during operation

	Preoperative	Skin incision	Primary tumor manipulation	After primary tumor resection	Post transfusion	Metastatic tumor manipulation	After metastatic tumor resection
HR (beats/min)	92	90	118	100	110	120	110
BP (mmHg)	120/70	100/61	170/107	110/60	120/64	150/90	100/57
CVP(mmHg)	10	8	7	6	9	8	5
CI [L/(min·m^2^)]	7.1	7.4	10.7	5.6	5.0	3.9	4.7
SVV(%)	6	7	10	25	16	10	14
PH		7.44	7.43	7.37			7.43
BE (mmol/L)		−0.8	−1.7	−3.0			−3.0
PaCO_2_(mmHg)		35	33	37			32
Hb (g/L)		124	124	102	112		106
K^+^(mmol/L)		4.8	4.4	3.9			4.7

*HR* heart rate, *BP* blood pressure, *SVV* stroke volume variation, *CVP* central venous pressure, *BE* base excess, *Hb* hemoglobin

When the girl arrived at the intensive care unit,norepinephrine infusion was continued at 0.12 μg/kg/min together with esmolol infusion at 100 mg/h to maintain the hemodynamics relatively stable with about 20% deviation from the baseline. In the first 24 h after the operation, the intropic support was continued and the patient received 2520 ml of crystalloids and colloids for blood volume expansion. But the norepinephrine infusion had to increased to 0.4 μg/kg/min under the guidance of consistent monitoring of BP(range,80–90/40-50 mmHg),HR(range,90-110beats/min)and CVP(range,5-8 mmHg). The coagulation studies was normal, but NT-proBNP was 844 pg/ml while her echocardiogram showed a deteriorated LVEF of 41% and mild left ventricular hypertrophy. Considered as the factor of insufficient blood volume, in the next 24 h, acetated Ringer solution (2600 ml),concentrated red blood cells(200 ml)and albumin (100 ml)had been rapid infused. Her arterial blood pressure slowly improved to 110/60 mmHg and the estimated LVEF improved to 50% with adequate urine output. All intraoperative monitoring was continued for 5 days before cardiovascular stability was conformed without the vasoactive drugs support, and she was discharged home by postoperative day 15.

## Discussion and conclusions

Pheochromocytomas and paragangliomas are rare tumors in children, multiple and extra-adrend paragangliomas are more common in children than in adults. Perioperative management should follow the same principles as for adults [5]. The symptoms of pheochromocytomas result from the abrupt release of large amount of catecholamines. The most commonly symptoms are palpitations,excessive sweating and headache [6]. Our patient initially presented with palpitation 、hypertension and dyspnea on exertion as a result of uncontrolled norepinephrine resease and catecholamine-induced cardiomyopathy, the released norepinephrine acts predominantly onα_1_ and β_1_ adrenoreceptors resulting in tachycardia and vasoconstriction.

Adrenergic α-blockers are traditionally applied to control blood pressure before surgical operation and to prevent hypertensive crisis during surgery. Phenoxybenzamine irreversibly bound to α_1_-and α_2_- adrenoreceptors has a pharmacological half-life of 24 h [7, 8]. Postural hypotension and reflex tachycardia are the most common side effects of phenoxybenzamine owing to the blockade of α_2_-receptors. [7, 8]. The Endocrine Society consensus guidelines recommend oral phenoxybenzamine or other α-adrenergic antagonists as the first-line agents for perioperative blood pressure control [9]. However, the routine use of phenoxybenzamine is controversial because there were also literatures indicated that several fast-acting vasoactive drugs could rapidly control intraoperative hemodynamic changes whenever necessary and preoperative α-blockade was not judged necessary. In a study on 96 patients undergoing pheochromocytoma resection with no mortality, a high preoperative arterial pressure level could not indicate intra-and post-operative hemodynamic instability [10]. In another study, Ulchaker et al. [11] compared patients who were or were not treated preoperatively with antihypertensive drugs, found that intraoperative mean blood pressure level were similar.

General anesthesia is favored in most reports as vasodilation associated with spinal anesthesia does not protect against the hemodynamic variability caused by catecholamine release during tumor resection [12]. In patients with pheochromocytoma, intubation and surgical incision are widely recognized periods of potential hemodynamic instability. In the case, preoperative volume expansion, adrenergic blockade as well as suitable anesthesia method provided relatively stable hemodynamics without the use of additional vasodilators.

The guidelines from the American College of Critical Care Medicine suggest the role of direct pulmonary artery monitoring in children with severe pulmonary hypertension to improve the outcome [13]. Its role in intraoperative management of pheochromocytoma resection for adults has been previously described, but its utility for pheochromocytoma resection in child with catecholamine-induced cardiomyopathy has not been described. In our case, direct pulmonary artery monitoring was not selected as tool for guilding volume resuscitation due to increased complications from device insertion and enough hemodynamic data gathered from other invasive monitoring (invasive artery blood pressure monitoring and central venous pressure monitoring).

The FloTrac/VigileoTM system (Edwards Life-Sciences, Irvine, CA, USA) has been introduced for more than ten years to use peripheral arterial waveform to calculate cardiac output and stroke volume variation (SVV). Spontaneous breathing, tidal volume<8 ml/kg when mechanical ventilation, severe arrhythmias, high pneumoperitoneum pressure, use of vasoactive agents and some other factors will affect the reliability and authenticity of FloTrac / Vigileo system measurement results [14]. The FloTrac technology based on arterial pressure waveform is now FDA-approved for use in adults but not in children. A review showed that dynamic variables got from the devise could not predict fluid responsiveness in pediatric patients [15]. But a recent study revealed that stroke volume variation (SVV) got from the FloTrac/VigileoTM monitoring system can reflect a change in blood volume during the blood removal and fluid replacement protocol in acute normovolemic hemodilution (ANH) in children [16]. Several studies suggest that the possible explanation is that the FloTracTM algorithm was validated in elderly patients with atherosclerotic vessels,and the vascular properties of atherosclerotic vessels are different from those of children’s vessels [17, 18]. Although arterial wall distensibility decreases significantly with age and declines fastest during the children’s first several years of life, the age at which they resemble those in adults is uncertain. And the authors believe that their results might be due to the study protocol, which involved recruiting relatively large children (2–7.5 years) [16]. In our case, we tried to observe the performance of FloTrac/VigileoTM monitoring system in reflecting the change in blood volume of the young patient,and we found that SVV rised significantly at each time point we made the judgment that the patient was in low blood volume status according to other hemodynamic parameters. As the patient was already nine years old, her arterial wall compliance might already resemble that in adults according to the explanation of the above study. And we need to observe more cases about the usage of the Flo-TracTM in children to obtain more convincing data.

The most important aspects in the management of a patient with paragangliomas and catecholamine-induced cardiomyopathy are the control of blood pressure liability while tumor manipulation and the maintenance of intravascular volume status. In the case, we prepared phentolamine,sodium nitroprusside and esmolol to control intraoperative hypertension as their characteristics of quick onset and short duration [19].

Severe hypotension may occur once the tumor is isolated and resected result from the abrupt cessation of catecholamine secretion and depleted blood volume [20, 21]. In order to prevent the severe hypotension, we used various quantities and types of fluids associated with vasoactive drugs, mainly norepinephrine to control hypotension following tumor withdrawal as the preoperative examination showed that the tumors mainly secreted norepinephrine.

Pheochromocytomas patient may have cardiomoyopathy owing to myocardial toxicity of high circulating catecholamines level [22]. It may cause hypertrophic or dilated cardiomyopathy and the cardiomyopathy can present at any age, even in childhood [23, 24]. Increased left ventricular end diastolic and systolic dimensions and volumes,reduced left ventricular ejection fraction(EF) and wall motion abnormalities are common documented echocardiographically in catecholamine-induced cardiomyopathy [22, 25]. However, the literatures on catecholamine cardiomyopathy are limited to case reports, with few reports of studies on children. Pheochromocytoma presenting as Takotsubo or inverted Takotsubo cardiomyopathy is newly recognized occurrence with increasing number of cases. They have been postulated that acute and reverted multiterritorial coronary microcirculation vasoconstriction was an important pathophysiological mechanism. When we meet patients diagnosed with acute coronary syndrome symptoms and show pronounced blood pressure variability but lack coronary artery stenosis or spasm in the clinic, pheochromocytoma-induced Takotsubo cardiomyopathy should be considered [26–30]. In our case, the patient’s preoperative transthoracic echocardiogram showed mild mitral regurgitation and left ventricular dilation(LVEDV,43 mm) with an estimated LVEF of 49%, then the diagnosis of catecholamine cardiomyopathy was comfirmed.

Paraganglioma resection is effective curative surgery for paraganglioma with catecholamine-induced cardiomyopathy. Catecholamine-induced cardiomyopathy can be reversed weeks after tumor resection is a well known phenomenon [25]. In a prospective, case-control study, Gaurav et al. [22] reported that after curative paraganglioma resection surgery, all the echocardiographic parameters(approximately 15%improvement in LV volumes)and serum NTpro-BNP levels(56% fall) improved markly at 1 week postoperatively. Sustained and continued improvements in serum NTpro-BNP and echocardiographic parameters were noticed at 3 and 6 months of follow-up. A literature search revealed only one case in which the catecholamine cardiotoxicity required heart transplant after removal of the catecholamine-secreting tumor [31].

There is no specific therapeutic approach for catecholamine-induced cardiomyopathy up to now. The perioperative management of catecholamine-induced cardiomyopathy should include lowering sympathetic activation by means of α-and β-adrenergic receptor blocker and diuretics administration in case of volume overload [32]. The emergency management of arrhythmia, pulmonary edema, and cardiogenic shock is based on conventional symptomatic treatment, such as antiarrhythmic and inotropes, vasoactive electrical shock and/or mechanical circulatory support.

## Abbreviations

BP: blood pressure
CVP: central venous pressure
HR: heart rate
ICU: intensive care unit
LV: left ventricle
LVEF: left ventricular ejection fraction
PET-CT: Positron Emission Computed Tomography

## References

[1] Renard J, Clerici T, Licker M (2011). Pheochromocytoma and abdominal paraganglioma. J Visc Surg.

[2] Guerrero MA, Schreinemakers JM, Vriens MR, Suh I, Hwang J, Shen WT, Gosnell J, Clark OH, Duh QY (2009). Clinical spectrum of pheochromocytoma. Journal of the American College of Surgeons.

[3] Kopetschke R, Slisko M, Kilisli A, Tuschy U, Wallaschofski H, Fassnacht M, Ventz M, Beuschlein F, Reincke M, Reisch N, Quinkler M (2009). Frequent incidental discovery of phaeochromocytoma:data from a German cohort of 201 phaeochromocytoma.European. Journal of Endocrinology.

[4] Lenders JW, Eisenhofer G, Mannelli M, Pacak K (2005). Phaeochromocytoma. Lancet.

[5] Naranjo J, Dodd S, Martin YN (2017). Perioperative Management of Pheochromocytoma. J Cardiothorac Vasc Anesth.

[6] Prejbisz A, Lenders JWM, Eisenhofer G (2011). Et al.Cardiovascular manifestations of phaeochromocytoma. J Hypertens.

[7] Pacak K (2007). Preoperative management of the pheochromocytoma patient. J Clin Endocrinol Metab.

[8] McMillian WD,Trombley BJ,Charash WE&Christian RC.Phentolamine continuous infusion in a patient with pheochromocytoma.Am J Health Syst Pharm 2011;68:130–134. 10.2146/ajhp090619 21200059

[9] Lenders JW, Duh QY, Eisenhofer G (2014). Pheochromocytoma and paraganglioma: an endocrine society clinical practice guideline. J Clin Endocrinol Metab.

[10] Lentschener C, Gaujoux S, Thillois JM, Duboc D, Bertherat J, Ozier Y, Dousset B (2009). Increased arterial pressure is not predictive of haemodynamic instability in patients undergoing adrenalectomy for phaeochromocytoma. Acta Anaesthesiologica Scandinavlca.

[11] Ulchaker JC, Goldfarb DA, Bravo EL, Novick AC (1999). Successful outcomes in pheochromocytoma surgery in the modern era. Journal of Urology.

[12] Bruynzeel H, Feelders RA, Groenland TH, van den Meiracker AH, van Eijck CH, Lange JF, de Herder WW, Kazemier G (2010). Risk factors for hemodynamic instability during surgery for pheochromocytoma. Journal of Clinical Endocrinology and Metabolism.

[13] Brierley J, Carcillo JA, Choong K (2009). Clinical practice septic shock :2007 update from the American College of Clinical Care Medicine. Crit Care Med.

[14] Slagt C, Malagon I, Groeneveld AB (2014). Systematic review of uncalibrated arterial pressure waveform analysis to determine cardiac output and stroke volume variation. Br J Anaesth.

[15] Gan H, Cannesson M, Chandler JR (2013). Predicting fluid responsiveness in children: a systematic review. Anesth Analg.

[16] Tadokoro T, Kakinohana M, Fukumoto C (2016). Usefulness of stroke volume variation to assess blood volume during blood removal for autologous blood transfusion in pediatric patients. Pediatric Anaesth.

[17] Teng S, Kaufman J, Pan Z (2011). Continuous arterial pressure waveform monitoring in pediatric cardiac transplant, cardiomyopathy and pulmonary hypertension patients. Intensive Care Med.

[18] Byon HJ, Lim CW, Lee JH (2013). Prediction of fluid responsiveness in mechanically ventilated children undergoing neurosurgery. Br J Anaesth.

[19] Lentschener C, Gaujoux S, Tesniere A, Dousset B (2011). Point of controversy:perioperative care of patients undergoing pheochromocytoma removal-time for a reappraisal?European Jounal of. Endocrinology.

[20] Grasselli G, Foti G, Patroniti N, Rona R, Periangeli MV, Pesenti A (2008). Extracorporeal cardiopulmonary support for cardiogenic shock caused by pheochromocytoma:a case report and literature review. Anesthesiology.

[21] Wu GY, Doshi AA, Haas GJ (2007). Pheochromocytoma induced cardiogenic shock with rapid recovery of ventricular function. Eur J Heart Fail.

[22] Agarwal G, Sadacharan D, Kapoor A, Batra A (2011). Cardiovascular dysfunction and catecholamine cardiomyopathy in pheochromocytoma patients and their reversal following surgical cure:Results of a prospective case-control study. Surgery.

[23] Aleksander Prejbisz,Jacques W.M.Lenders,Graeme Eisenhofer,Andrzej Januszewicz .Cardiovascular manifestations of phaeochromocytoma.Journal of Hypertension 2011,29:2049–2060. 10.1097/HJH.0b013e32834a4ce9 21826022

[24] Kim J, Reutrakul S, Davis DB, Kaplan EL, Refetoff S (2004). Multiple endocrine neoplasa 2A syndrome presenting as peripartum cardiomyopathy due to catecholamine excess. Eur J Endocrinol.

[25] Takizawa M, Kobayakawa N, Uozumi H, Yonemura S, Kodama T, Fukusima K, Takeuchi H, Kaneko Y, Kaneko T, Fujita K, Honma Y, Aoyagi T (2007). A case of transient left ventricular ballooning with pheochromocytoma,supporting pathogenetic role of catecholamines in stress-induced cardiomyopathy or takotsubo cardiomyopathy. Int J Cardiol.

[26] Jindal V, Baker ML, Aryangat A, Wittlin SD, Bisognano JD, Richter HS (2009). Pheochromocytoma: presenting with regular cyclic blood pressure and inverted Takotsubo cardiomyopathy. J Clin Hypertens.

[27] Lassnig E, Weber T, Auer J, Nomeyer R, Eber B (2009). Pheochromocytoma crisis presenting with shock and tako-tsubo-like cardiomyopathy. Int J Cardiol.

[28] Zielen P, Klisiewicz A, Januszewicz A, Prejbisz A, Kabat M, Peczkowska M (2010). Pheochromocytoma-related ‘classic’ takotsubo cardiomyopathy. J Hum Hypertens.

[29] Galiuto L, De Caterina AR, Porfidia A, Paraggio L, Barchetta S, Locorotondo G (2010). Reversible coronary microvascular dysfunction: a common pathogenetic mechanism in apical ballooning or Tako-Tsubo syndrome. Eur Heart J.

[30] Eugene A, Hessel MD (2016). Takotsubo cardiomyopathy and its relevance to anesthesiology: a narrative review. J Can Anesth.

[31] Wester T, Franklin A, Donahue BS (2015). Perioperative Management of a Pediatric Patient with catecholamine-induced cardiomyopathy undergoing laparoscopic Paraganglioma excision requiring biventricular assist device support. J Cardio thorac Vasc Anesth.

[32] De Miguel V, Arias A, Paissan A (2014). Catecholamine-induced myocarditis in pheochromocytoma. Circulation.

